# Characterization of two heparan sulphate-binding sites in the mycobacterial adhesin Hlp

**DOI:** 10.1186/1471-2180-8-75

**Published:** 2008-05-15

**Authors:** Michelle I Portugal, Adriane R Todeschini, Cristiana S de Lima, Carlos AM Silva, Ronaldo Mohana-Borges, Tom HM Ottenhoff, Lucia Mendonça-Previato, Jose O Previato, Maria CV Pessolani

**Affiliations:** 1 Laboratório de Microbiologia Celular, Instituto Oswaldo Cruz, FIOCRUZ, Av. Brasil 4365, Manguinhos, Rio de Janeiro, RJ 21045-900, Brazil; 2 Laboratório de Glicobiologia, Instituto de Biofísica Carlos Chagas Filho, Universidade Federal do Rio de Janeiro, Av. Brigadeiro Tropowsky, Rio de Janeiro, RJ, 21949-900, Brazil; 3 Laboratório de Genomica Estrutural, Instituto de Biofísica Carlos Chagas Filho, Universidade Federal do Rio de Janeiro, Av. Brigadeiro Tropowsky, Rio de Janeiro, RJ, 21949-900, Brazil; 4 Department of Immunohematology and Blood Transfusion, Leiden University Medical Center, Building 1 E3-Q, P.O. box 9600, 2300 RC – Leiden, The Netherlands

## Abstract

**Background:**

The histone-like Hlp protein is emerging as a key component in mycobacterial pathogenesis, being involved in the initial events of host colonization by interacting with laminin and glycosaminoglycans (GAGs). In the present study, nuclear magnetic resonance (NMR) was used to map the binding site(s) of Hlp to heparan sulfate and identify the nature of the amino acid residues directly involved in this interaction.

**Results:**

The capacity of a panel of 30 mer synthetic peptides covering the full length of Hlp to bind to heparin/heparan sulfate was analyzed by solid phase assays, NMR, and affinity chromatography. An additional active region between the residues Gly46 and Ala60 was defined at the N-terminal domain of Hlp, expanding the previously defined heparin-binding site between Thr31 and Phe50. Additionally, the C-terminus, rich in Lys residues, was confirmed as another heparan sulfate binding region. The amino acids in Hlp identified as mediators in the interaction with heparan sulfate were Arg, Val, Ile, Lys, Phe, and Thr.

**Conclusion:**

Our data indicate that Hlp interacts with heparan sulfate through two distinct regions of the protein. Both heparan sulfate-binding regions here defined are preserved in all mycobacterial Hlp homologues that have been sequenced, suggesting important but possibly divergent roles for this surface-exposed protein in both pathogenic and saprophic species.

## Background

Leprosy and tuberculosis constitute age-old infectious diseases that have affected human beings for millennium. Leprosy, caused by *Mycobacterium leprae*, continues to be a significant public health problem in several developing countries, including Brazil, and is responsible for the legacy of millions of individuals with permanent physical deformities [1]. On the other hand, tuberculosis, caused by *Mycobacterium tuberculosis *has most likely killed more human beings than any other disease in history, at an average of 4 individuals per minute worldwide [2]. One major reason for the failure to eradicate these diseases may be closely related to the absence of effective vaccines since BCG, the only one available against both leprosy and tuberculosis, has displayed highly variable protection rates around the world [3]. Mycobacteria are intracellular pathogens that preferentially infect mononuclear phagocytes although other cell types such as epithelial and endothelial cells are also colonized during disease dissemination [4]. *M. leprae*, also found inside Schwann cells of the peripheral nervous system, is responsible for the nerve damage observed in leprosy [5]. Mycobacteria are Gram-positive bacteria presenting a very complex and unique cell wall structure, which seems to play a major role in their pathogenesis. Deciphering the mechanisms implicated in mycobacterial pathogenesis remains a major challenge for leprosy and tuberculosis research, which will hopefully lead to the development of new, more effective prophylactic and/or therapeutic strategies.

During infection, a critical early event is the adherence of the microorganism to the target tissues within the host. Adhesion is accomplished by specific molecular interactions involving adhesins on the bacterial surface and receptors on the surface of the host cell. The histone-like Hlp protein is a positively-charged, surface-exposed molecule recently implicated in the attachment of pathogenic mycobacteria to host cells. This protein was initially described as a laminin-binding protein (LBP) involved in *M. leprae*-Schwann cell interaction. [6,7]. More recently Hlp has been shown to also play a major role in mediating the adhesion of mycobacteria to epithelial respiratory cells by interacting with proteoglycan-containing receptors such as heparan sulfate and hyaluronic acid [8,9].

Mycobacterial Hlp, roughly twice the size of other bacterial histone-like proteins, is a highly conserved protein shared by all mycobacterial species. The N-terminal half of mycobacterial Hlp containing the prokaryotic DNA-binding motif shares significant homology with the histone-like HU proteins found in *Escherichia coli *and the HB proteins in *Bacillus subtilis*, respectively. The C-terminal half of mycobacterial Hlp, absent in most bacteria, has an unusual amino acid composition owing to a high alanine, lysine, and proline content resembling the C-terminal region of eukaryotic class H1 histones [10]. Initial studies by Aoki *et al *[9] have mapped the heparin-binding site of Hlp (so-called MDP1) in the N-terminal region between Thr31 and Phe50 overlapping the previously defined DNA-binding region [11]. However, after testing the truncated recombinant Hlp molecules corresponding to the N-terminal (rHlp-N) and the C-terminal (rHlp-C) domains of the protein, we recently found that the interaction of Hlp/LBP with laminin and heparin was for the most part mediated by the C-terminal domain of the protein. Moreover, the same domain was found to be involved in Hlp/LBP-mediating bacterial binding to human Schwann cells [12]. It is clear that additional studies are needed to more precisely define the interacting regions of Hlp/LBP with glycosaminoglycans (GAG).

In the present report, the interaction of Hlp with heparan sulfate was further investigated by using a panel of 30-mer synthetic peptides covering the full length of the protein and nuclear magnetic resonance (NMR). The resulting data indicate that Hlp interacts with heparan sulfate through two distinct regions located, respectively, in the N-terminal and C-terminal domains of the protein.

## Results

### Binding of Hlp synthetic peptides to heparan sulfate

**Figure 1:**
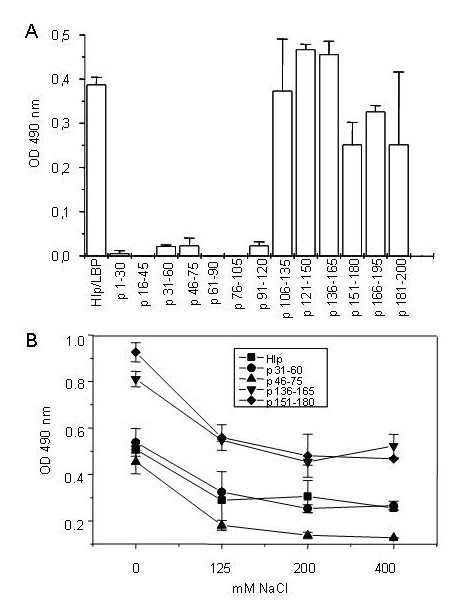
**Hlp peptides corresponding to the C-terminal domain bind heparan sulfate**. (A) Microtiter-plate wells coated with 50 μL of 0.1 μM Hlp or 50 μL of 0.65 μM individual 30-mer synthetic peptides covering the entire sequence of Hlp were incubated with biotinylated heparan sulfate in phosphate buffer 10 mM, pH 7.2. Heparan sulfate binding was measured by adding streptavidin peroxidase to the wells and expressed in absorbency units at 490 nm. The data are expressed as mean ± SD of a representative experiment from five independent ones performed in duplicate. (B) Microtiter-plate wells were coated with Hlp or peptides p31–60, p136–165, and p151–180 and biotinylated heparan sulfate was added in the presence of increasing concentrations of NaCl. Heparan sulfate binding was quantified as above.

In a previous study, truncated recombinant Hlp molecules were tested and it was demonstrated that the highly positive C-terminal region of the protein is involved in its binding to extracellular matrix components and its capacity to act as an adhesin [12]. To refine and confirm these data, a panel of overlapping 30-mer synthetic peptides covering the entire sequence of Hlp was immobilized in microtiter-plate wells. The peptides were, then, incubated with biotinylated heparin or heparan sulfate in phosphate buffer 10 mM pH 7.2 and their binding capacity was monitored by the addition of streptavidin peroxidase. The list of Hlp peptides with their corresponding amino acid sequences and pI shown in Table 1. As shown in Figure 1A, heparan sulfate bound to peptides covering the C-terminal region of the protein. Among the peptides derived from the N-terminal region, detectable binding of biotinylated heparan sulfate was only observed in wells coated with p31–60 and p46–75. Although the signal with these peptides was low, this was a consistent result observed in six independent experiments. An identical binding pattern was observed when biotinylated heparin was used instead of heparan sulfate (data not shown). The binding of the peptides covering the C-terminal region is in agreement with previous results demonstrating that rHlp-C (from Ala110 to Lys200), but not rHlp-N (covering the first 109 Hlp amino acids), was able to bind heparan sulfate in a similar solid-phase assay [12]. Moreover, the binding of p31–60 is in agreement with the Hlp heparin-binding region between Thr31 and Phe50 defined by Aoki *et al*, 2004 [9]. However, the binding of p46–75 suggested an additional binding region.

**Table 1:** Amino acid sequence and pI values of Hlp synthetic peptides. Basic amino acids are in bold.

**Peptide**	**Amino acid sequence**	**pI**
**p1**	^**1**^MN**K**AELIDVLTQ**K**LGSD**RR**QATAAVENVVD^**30**^	**5.00**
**p2**	^**16**^SD**RR**QATAAVENVVDTIV**R**AV**HK**GDSVTIT^**45**^	**6.48**
**p3**	^**31**^TIV**R**AV**HK**GDSVTITGFGVFEQ**RRR**AA**R**VA^**60**^	**11.83**
**p4**	^**46**^GFGVFEQ**RRR**AA**R**VA**R**NP**R**TGETV**K**V**K**PTS^**75**^	**12.01**
**p5**	^**61**^**R**NP**R**TGETV**K**V**K**PTSVPAF**R**PGAQF**K**AVVA^**90**^	**11.73**
**p6**	^**76**^VPAF**R**PGAQF**K**AVVAGAQ**R**LPLEGPAV**KR**G^**105**^	**11.72**
**p7**	^**91**^GAQ**R**LPLEGPAV**KR**GVATSAA**KK**AAI**KK**AP^**120**^	**11.22**
**p8**	^**106**^VATSAA**KK**AAI**KK**APV**KK**ALA**KK**AAT**K**APA^**135**^	**10.90**
**p9**	^**121**^V**KK**ALA**KK**AAT**K**APA**KK**AV**K**APA**KK**ITTAV^**150**^	**10.95**
**p10**	^**136**^**KK**AV**K**APA**KK**ITTAV**K**VPA**KK**AT**K**VV**KK**VA^**165**^	**11.00**
**p11**	^**151**^**K**VPA**KK**AT**K**VV**KK**VAA**K**APV**RK**ATT**R**ALA**K**^**180**^	**10.70**
**p12**	^**166**^A**K**APV**RK**ATT**R**ALA**KK**AAV**KK**APA**KK**VTAA^**195**^	**12.06**
**p13**	^**181**^**K**AAV**KK**APA**KK**VTAA**KR**G**RK**^**200**^	**12.05**

### Effect of salt concentrations on the binding of Hlp peptides to heparan sulfate in microplate assays

All peptides that showed heparan sulfate-binding activity are rich in basic amino acid residues. To investigate whether Hlp-heparin/heparan sulfate interactions are mediated by electrostatic forces involving the negatively charged sulfate and carboxyl groups of heparin and heparan sulfate with the positively charged residues of Hlp, binding assays were performed in the presence of increasing concentrations of NaCl. As can be seen in Figure 1B, for p31–60, p46–75, p136–165, and p151–180 and the entire Hlp protein, significant binding inhibition was achieved with increasing concentrations of NaCl, indicating the involvement of electrostatic forces in these interactions.

### Binding of p31–60, p136–165, and p151–180 to heparin-Sepharose

**Figure 2:**
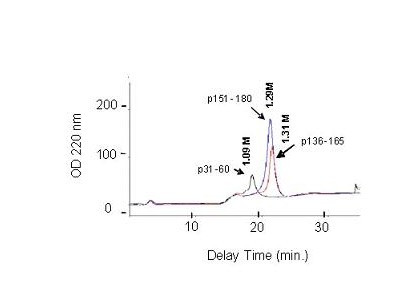
**Elution profiles of p31–60, p136–165, and p151–180 from a Heparin-Sepharose affinity colunm**. Individual peptides bound to the column were eluted with a 0 – 2 M gradient of NaCl. Peptides p31–60, p136–165, and p151–180 were eluted, respectively, at 1.09, 1.31, and 1.29 M NaCl.

The binding capacity of p31–60, p136–165, and p151–180 was also analyzed by affinity chromatography on a column of heparin-Sepharose. Individual peptides were applied to a heparin-Sepharose column and subsequently eluted with a 0–2 M NaCl gradient. Figure 2 shows the elution profile and the salt concentration necessary for elution of each peptide. Peptides p31–60, p36–165, and p151–180 bound to the column and were eluted at 1.09, 1.31, and 1.29 M NaCl, respectively, confirming their heparin-binding capacity.

### Analysis of the interaction of Hlp peptides with heparan sulfate by STD-NMR

To further elucidate the basis of Hlp and glycosaminoglycan interaction, the complexes between the peptides p16–45, p31–60, p46–75, p136–165, p151–180, and heparan sulfate were studied using Saturation Transfer difference – (STD)-NMR experiments. This technique allows for the identification of protons from a ligand molecule in contact with a macromolecule. Resonances of the oligosaccharide were selectively saturated and the spectrum was subtracted from a reference spectrum from which the heparan sulfate was not saturated. Lastly, the enhancements were observed in the difference (STD) spectrum [13,14]. Protons of the ligand peptide, which were in close contact with heparan sulfate, received the highest degree of saturation and were easily identified within the STD spectrum.

**Figure 3:**
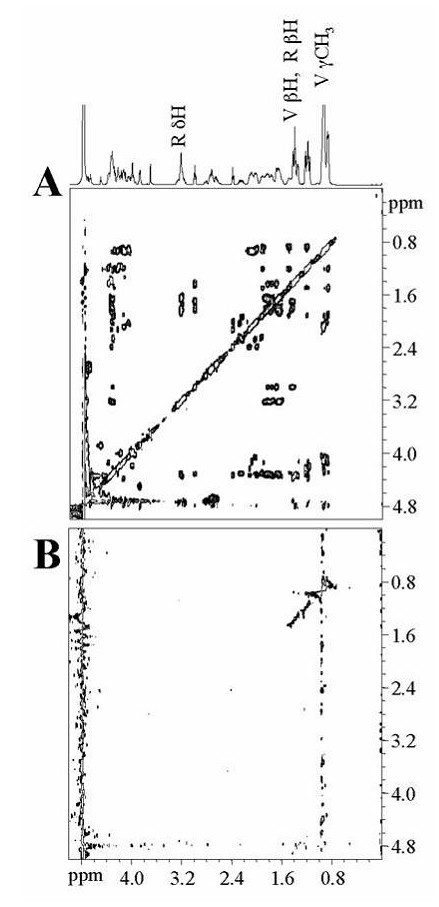
**STD-TOCSY of p16–45 in presence of heparan sulfate**. (A) Reference TOCSY spectrum of p16–45 (2 mM) in the presence of heparan sulfate (10 μM). (B) STD-TOCSY spectrum. Spectra were recorded in PBS-D_2_O, pH 7.6, 25°C with mixing time of 66 ms, 32 scans per *t*1 increment. 200 increments were collected in an interlaced mode for on or off pre-saturation.

**Figure 4:**
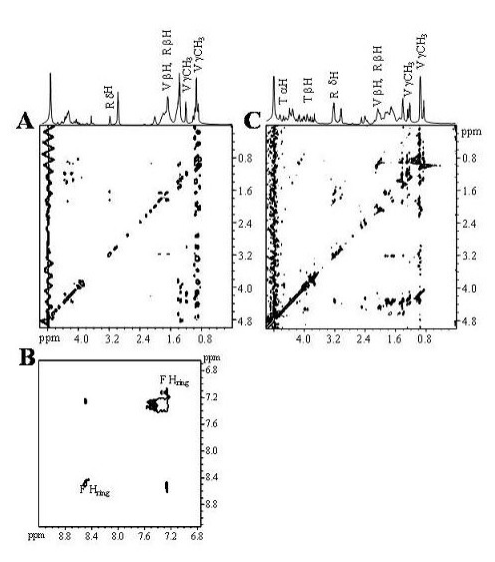
**Association of peptides p31–60 and p46–75 with heparan sulfate**. (A) STD-TOCSY of p31–60 (2 mM) in the presence of heparan sulfate (10 μM). (B) STD-TOCSY spectrum of the aromatic region. (C) STD-TOCSY of p46–75 (2 mM) in the presence of heparan sulfate (10 μM). Spectra were recorded as in Figure 3.

NMR experiments confirmed the heparan-sulfate binding capacity of p31–60, p46–75, p136–165, p51–180, and the inability of p16–45 to do so. Figure 3 shows the reference TOCSY spectrum (Figure 3A) of heparan sulfate (100 μg) in the presence of 2 mM p16–45. The difference in the Total Correlation Spectroscopy – (TOCSY)-STD spectrum obtained (Figure 3B) shows no signal, indicating that peptide p16–45 does not bind to heparan sulfate, thus providing a negative control with no specific interaction with the macromolecule. Nevertheless, Figure 4 shows a clear interaction between peptide p31–60 and heparan sulfate. Figure 4A shows the cross peaks of the ligand protons involved in the formation of the complex. A close analysis of the STD spectrum shows a correlation among the δH at 3.18 ppm, βH and γH at 1.85, and 1.65 ppm, respectively, suggesting that at least one of the Arg residues is in close contact with heparan sulfate. In addition, it is evident that the magnetization is also transferred to the γCH_3 _of Val. Figure 4B provides evidence of the interaction of Phe residues with heparan sulfate. From the cross peaks at 3.19, 3.22 ppm (δH) and those at 1.89 (βH) and 1.63 ppm (γH) assigned in Figure 4C, it is evident that heparan sulfate binds to the Arg (R) residues of p46–75. The interaction of the amino acid Val (V) is evidenced by signals at 0.89 and 1.41 ppm. Furthermore, the interaction of at least one of the Thr (T) residues is evidenced by the resonances arising from γCH_3 _(1.29 ppm), βH (3.96 ppm), and αH (4.15 ppm).

**Figure 5:**
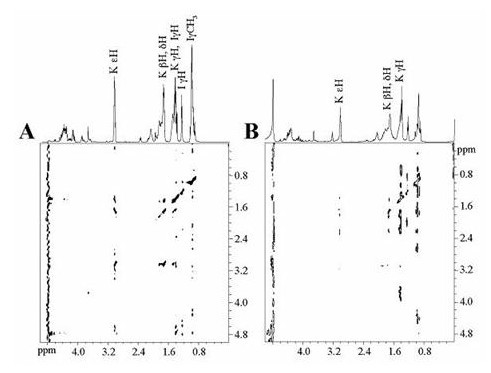
**Association of peptides p136–165 and p151–180 with heparan sulfate**. (A) STD-TOCSY of p136–165 (2 mM) in association with heparan sulfate (100 μg). (B) STD-TOCSY of p151–180 (2 mM) in the presence of heparan sulfate (10 μM). Spectra were recorded as in Figure 3.

Figure 5A shows that the peptide p136–165 binds to heparan sulfate. The STD spectrum demonstrates that the amino acids involved in contact with the macromolecule were Lys (3.02, 2.99, 1.73, and 1.38 ppm), and the hydrophobic residue Ile146 (γCH3, 0.96 ppm, and γH, 1.23 and 1.38 ppm). Finally, the STD-TOCSY spectrum in Figure 5B shows the amino acid Lys from the peptide p151–180 receiving the highest saturation transferance from heparan sulfate.

## Discussion

It is well established that adhesion of bacteria to target host tissues is required for colonization and subsequent development of disease [15]. Since adherence is a key step in microbial pathogenesis, the use of anti-adhesion therapy and anti-adhesion immunity has emerged as an attractive approach toward the development of new tools to control infectious diseases [16]. In order to develop anti-adhesive drugs, including adhesin-based vaccines, a detailed understanding of the mechanisms by which microorganisms initiate host cell colonization is necessary. It has been reported that Hlp is a pivotal adhesion molecule in the context of mycobacteria interaction with host cells. It was initially described as a laminin-binding protein whose function was to mediate *M. leprae *adhesion to Schwann cells in conjunction with PGL-I, another laminin-binding molecule present on the surface of *M. leprae *[6,7,17]. Later on, the capacity of Hlp to bind heparin/heparan sulfate became evident along with the relevance of this interaction in the context of *M. tuberculosis *attachment to respiratory epithelial cells and the potential involvement of this protein in the initial events of host colonization [8,9,12].

Due to the emergence of Hlp as a key component in mycobacterial pathogenesis, a detailed analysis of the molecular regions involved in the interaction with extracellular matrix components was initiated. The first investigation in this direction reported that the region between Thr31 and Phe50 was responsible for the interaction of Hlp with heparin [9]. However, we recently showed that a fragment of the protein corresponding to the last 91 amino acids was additionally able to bind heparan sulfate [12]. To further define the heparan sulfate-binding sites of Hlp in the present study, a panel of 30-mer synthetic peptides derived from the entire sequence of this adhesin was used. Three distinct assays were employed to map the regions of Hlp/LBP involved with heparin/heparan sulfate interaction: solid phase assays in microplates, heparin-Sepharose affinity chromatography, and NMR. The results obtained indicate that Hlp interacts with heparin/heparan sulfate in two distinct regions: one located at the N-terminal half of the protein between residues 31 and 60, and the other corresponding to the entire C-terminal half of the protein.

The features of Hlp heparan sulfate-binding regions herein defined are typical of heparin-binding consensus sequences. As such, they were expected to interact with GAGs (for review, see [18]. The definition of the first site was based on the capacity of peptides p31–60 and p46–75 to bind heparin and heparan sulfate using three different assays. These overlapping peptides share the QRRRAAR sequence (residues 52 to 59) that fits perfectly into the heparin-binding consensus XBBBXXB sequence (where X is an hydropathic amino acid and B is a basic residue) previously defined by Cardin and Weintraub [18]. NMR experiments confirmed that p31–60 and p46–75, but not p16–45, were able to bind heparan sulfate. Moreover, peptide p31–60 was also able to bind tightly to a heparin-agarose column with elution only occurring at high salt concentrations.

A second GAG-binding region was characterized at the Hlp C-terminal. The 30-mer synthetic peptides p91–120, p106–135, p121–150, p136–165, p151–180, p166–195, and the 20-mer peptide p181–200 covering the C-terminal region of *M. leprae *Hlp were also able to bind heparin/heparan sulfate in solid phase assays. Two of these peptides, p136–165 and p151–180, were also tested for their capacity to bind heparin and heparan sulfate in affinity chromatography and NMR, respectively. As a result, their GAG-binding activity was confirmed. All peptides of this region are rich in positively charged residues (from 7 to 11 residues per peptide, mainly Lys), which occur singly or in clusters of two, mostly intercalated with Ala/Val residues. This same spacing pattern of basic amino acids has been found in several heparin-binding domains and seems to facilitate formation of ion pairs with spatially-defined sulfo- or carboxyl-groups in GAGs [19].

Direct NMR observation of the side chain proton resonances that are perturbed upon heparin binding has proven to be a powerful method of identifying the amino acids involved in protein-GAG interaction [20]. However, to our knowledge, this is the first time that STD-NMR has been applied to study the interaction of a ligand (a synthetic peptide) with a nonproteic macromolecule (heparan sulfate). Analysis of the interaction of p31–60, p46–75, p136–165, and p151–180 with heparan sulfate by STD-NMR led the way to identifying Arg, Val, Thr, Phe, Lys, and Ile as the amino acid residues directly involved in Hlp-GAG interaction.

**Figure 6:**
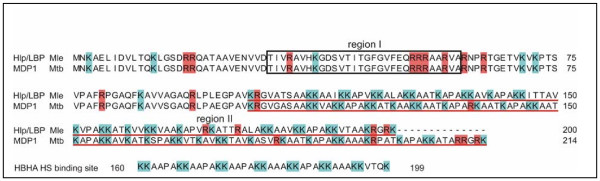
**Protein sequence alignment of Hlp from *M. leprae*, and MDP1 and HBHA from *M. tuberculosis***. The sequence of Hlp and MDP1 surrounded by the black straight-lined box corresponds to the heparin-interacting region (region I) proposed in this study. The sequence underlined by a red line represents the other heparin-interacting region (region II). The heparin-binding domain of *M. tuberculosis *HBHA is also shown on the bottom. The sequence alignment was prepared via the Jalview Java Alignment Editor version 2.2 [26].

The heparin-binding regions for *M. leprae *Hlp herein described only coincide partially with the one previously reported in *M. bovis *Hlp (so-called MDP1) [9]. These authors indicated that the sequence between Thr31 and Phe50 was the only heparin-binding site present in MDP1, excluding the cluster of Arg53, Arg54, Arg55, and Arg57 as well as the heparin-binding C-terminal domain of the protein. Their finding was based solely on solid phase assays in microplates using 20-mer synthetic peptides covering the entire length of the protein; and no additional experiments based on different techniques were performed to confirm these results. Moreover, the discrepancy between the Hlp heparin-binding sites cannot be explained by the fact that the Hlp proteins analyzed in both studies originated from different mycobacterial species. As shown in Figure 6, *M. leprae *and *M. bovis *BCG Hlp are 84% identical in their amino acid sequences. Actually, a multiple sequence alignment shows that the N-terminal heparin-binding region described in this study in addition to the one defined by Aoki *et al *[9] are conserved in all mycobacterial Hlp homologues sequenced to date (data not shown). Variations in Hlp sequences among mycobacterial homologues are mostly located at the C-terminal region even though the features recognized as important for heparin binding seem to be preserved across the species.

In summary, based on this study and previous observations [9,12], we propose that there are two distinct heparan sulfate-binding regions in Hlp: region I, from Thr31 to Ala60 and region II, spanning Lys103 to Lys200 (Figure 6). The identification of Arg in region I and Lys in region II confirms the critical participation of positively charged residues and electrostatic forces in mediating GAG-protein interaction. Moreover, NMR observations showed the direct involvement of Val, Thr, and Phe in region I and Ile in region II, indicating that specific nonionic interactions also take part in Hlp-heparan sulfate binding. As has been observed in other GAG-protein interactions, it is almost certain that these residues interact with N-acetyl and hydroxyl groups in heparan sulfate through hydrophobic interactions and hydrogen bonding, respectively, [18].

Reinforcing the role of the highly positive C-terminal domain in the interaction of Hlp with heparin/heparan sulfate, the heparin binding hemagglutinin (HBHA), another adhesin implicated in the interaction of mycobacteria with epithelial cells [21], also contains lysine-rich motifs in the C-terminal domain (from Lys161 to Lys199_; _Figure 6) that have been shown to mediate bacterial adherence via interaction with heparan sulfate-containing proteoglycans [22,23]. HBHA appears, however, to interact with heparin/heparan sulfate more weakly than Hlp [9], suggesting it plays a secondary role in adhesion. Indeed, as shown in this study, Hlp displays an expanded C-terminal GAG-binding domain and an additional heparin-binding region at the N-terminal domain when compared to HBHA, which may explain the higher affinity of Hlp to GAGs (Figure 6).

## Conclusion

The results obtained indicate that Hlp interacts with heparin/heparan sulfate in two distinct regions: one located at the N-terminal half of the protein between residues 31 and 60, and the other corresponding to the entire C-terminal half of the protein. Both heparan sulfate-binding regions are preserved in all mycobacterial Hlp homologues that have been sequenced, suggesting important but possibly divergent roles for this surface-exposed protein in both pathogenic and saprophic species. Due to the emergence of Hlp as a key component in mycobacterial pathogenesis, and the potential involvement of this protein in the initial events of host colonization, a detailed analysis of the molecular regions involved in the interaction with extracellular matrix components may contribute to the development of new prophylaxis and therapeutic interventions in mycobacterial diseases based on this adhesin.

## Methods

### *M. leprae *Recombinant Hlp and Peptides

Recombinant (r) *M. leprae *Hlp was obtained, as previously described [7]. Twelve 30-mer peptides, overlapping by fifteen amino acids and covering Met1 to Ala195 of *M. leprae *Hlp and one 20-mer peptide, corresponding to Lys181 to Lys200 of the protein, were synthesized by using solid-phase pin technology (Mimotopes, San Diego, CA, USA). Each peptide was dissolved in distilled water at a concentration of 10 mg/mL and stored frozen at -20°C until use.

### Solid-phase binding assays

Wells of a 96-well of polystyrene microtiter-plates (Corning, New York, NY, USA) were coated with 50 μL of Hlp (0.1 μM) or synthetic peptides (0.65 μM) in 0.1 M carbonate buffer pH 9.6. Plates were incubated overnight at 4°C. The wells were then washed with 10 mM of phosphate buffer pH 7.2 and blocked for 2 h with 200 μL of phosphate buffer-2% bovine serum albumin (BSA) at room temperature. Upon washing with phosphate buffer/0.05% Tween 20, 50 μL of 25 or 50 μg/mL of biotinylated heparin (Sigma) or 10 μg/mL of heparan sulfate (Sigma) in phosphate buffer with increasing NaCl concentration (0, 125, 200 and 400 mM) were added to the wells and incubation was performed at room temperature for 2 h. The wells were rinsed with phosphate buffer/Tween 20 and incubated with 50 μL of streptavidin-peroxidase (Pierce, Rockford, IL, USA) at 0.5 μg/mL. Peroxidase activity was revealed with hydrogen peroxide and O-phenylenediamine (OPD). The reaction was stopped with HCl and read at 490 nm using an automatic microplate scanning spectrophotometer (SpectraMAX 190; Molecular Devices Corp, Sunnyvale, CA, USA).

### NMR Experiments

Peptides p16–45, p31–60, p46–75, p136–165, p151–180 (2 mM final concentration), and heparan sulfate (10 μM) were dissolved in deuterated phosphate buffered saline (PBS), pH 7.6 (not correct for isotope effects). NMR spectra were obtained at a temperature probe of 25°C on a Bruker DMX 600 equipped with a 5-mm triple resonance probe.

### Saturation Transfer Difference (STD)

TOCSY-STD spectra correspond to a modified TOCSY sequence [24,25]. Spectra were recorded with a mixing time of 66 ms, 32 scans per t_1 _increment with pre-saturation on or off for 2 s. The on-resonance irradiation at the heparan sulfate was applied at a chemical shift of 5.5 ppm (where protein signals from all peptides were not observed). Off-resonance irradiation was applied at 30 ppm (where no signals were observed). Samples containing only the peptides were used as controls and did not show any STD effect, since the resulting diference spectrum did not contain any signal for the peptide. Two hundred t_2 _increments were collected in an interlaced mode after every on- and off-irradiation spectra to minimize artifacts arising from temperature and magnet instability. Prior to subtraction, both spectra were identically processed and phased. The acquisition time for the two-dimensional experiments was typically 16 h. The spectra were apodized with a square cosine bell function in both dimensions and zero-filled twice.

## Authors' contributions

MIP, ART, CSL and CAMS performed the experiments, the data analysis, interpreted the results and drafted the manuscript. MCVP, JOP and RMB participated in the design of the study, in evaluation of the results and in revision of the manuscript. THMO and LMP discussed the results and critically read the manuscript. All authors read and approved the final manuscript.
